# Medical School of Fairfield*Circular and Catalogue of the Faculty and Students of the College of Physicians and Surgeons of the Western District of the State of New York, in Fairfield, Herkimer county, 1836–7.

**Published:** 1837

**Authors:** 


### Art. III.—Medical School of Fairfield.*

Great as may be the distance between Cherry Valley and the Valley of the Mississippi, not a few of our readers are interested in the medical school which the former embosoms. The great northern wave of immigration annually brings into the states of Ohio, Indiana, Illinois and Michigan, many *alumni* of the *comparatively* old institution at Fairfield ; and to them we would say, that from the catalogue before us, their *Alma Mater* seems to be healthy and prosperous. The number of last year's class was 164 ; of whom 30 received diplomas in February last. So small a dividend indicates that the Faculty are not disposed to divide any of their capital stock.

* Circular and Catalogue of the Faculty and Students of the College of Physicians and Surgeons of the Western District of the State of New York, in Fairfield, Herkimer county, 1836-7.

It is creditable to the professors of Fairfield, (several of whom are not unknown to fame,) that they are able in a village to attract larger classes than their brethren of the *great* city, who have over them so many natural and statistical advantages.

We observe a recommendation to students to attend the lectures of the institution at the *beginning* of their studies. Why do not students every where commence their studies with a course of lectures, especially on Anatomy,—special, general, and morbid,—Physiology, Chemistry, Pharmacy, and the Natural History of Medicines? We venture to affirm, that more progress would be made in one winter, by a pupil who should pursue this course (taking care to dissect at the same time,) than by twelve months of the most laborious office study. Why do not students realize in anticipation, that the time devoted to Anatomy and Chemistry, by books alone, is almost thrown away? Why do not fathers open their eyes to this undeniable fact? In all our schools a student has to pay but twice for his tickets, and would not therefore increase his expenses on the plan here proposed, except by travelling to and from the institution. Unless when boarded and lodged in his father's house, he could live as cheap near a medical school, as in the neighborhood of a private preceptor. Even private preceptors would find their advantage in what is here recommended, as they could carry forward their pupils through the higher branches with far greater facility, after they had gotten an introduction to the lower, by the aid of demonstrations and experiments, than without such preparation. D.

